# Two-Point Orientation Discrimination Versus the Traditional Two-Point Test for Tactile Spatial Acuity Assessment

**DOI:** 10.3389/fnhum.2013.00579

**Published:** 2013-09-13

**Authors:** Jonathan Tong, Oliver Mao, Daniel Goldreich

**Affiliations:** ^1^Department of Psychology, Neuroscience & Behaviour, McMaster University, Hamilton, ON, Canada

**Keywords:** tactile perception, somatosensory discrimination, reliability and validity, neurological examination, psychophysics, sensory testing, spatial acuity

## Abstract

Two-point discrimination is widely used to measure tactile spatial acuity. The validity of the two-point threshold as a spatial acuity measure rests on the assumption that two points can be distinguished from one only when the two points are sufficiently separated to evoke spatially distinguishable foci of neural activity. However, some previous research has challenged this view, suggesting instead that two-point task performance benefits from an unintended non-spatial cue, allowing spuriously good performance at small tip separations. We compared the traditional two-point task to an equally convenient alternative task in which participants attempt to discern the orientation (vertical or horizontal) of two points of contact. We used precision digital readout calipers to administer two-interval forced-choice versions of both tasks to 24 neurologically healthy adults, on the fingertip, finger base, palm, and forearm. We used Bayesian adaptive testing to estimate the participants’ psychometric functions on the two tasks. Traditional two-point performance remained significantly above chance levels even at zero point separation. In contrast, two-point orientation discrimination approached chance as point separation approached zero, as expected for a valid measure of tactile spatial acuity. Traditional two-point performance was so inflated at small point separations that 75%-correct thresholds could be determined on all tested sites for fewer than half of participants. The 95%-correct thresholds on the two tasks were similar, and correlated with receptive field spacing. In keeping with previous critiques, we conclude that the traditional two-point task provides an unintended non-spatial cue, resulting in spuriously good performance at small spatial separations. Unlike two-point discrimination, two-point orientation discrimination rigorously measures tactile spatial acuity. We recommend the use of two-point orientation discrimination for neurological assessment.

## Introduction

Two-point discrimination (2PD) has been used to measure tactile spatial acuity ever since E. H. Weber published his seminal work on the sense of touch, *De Tactu*, in 1834 (Weber, 1996). The 2PD task is convenient to apply and is widely used to assess cutaneous innervation and central somatosensory function (Dellon, 1981; American Society for Surgery of the Hand, 1983; Van Boven and Johnson, 1994; Lundborg and Rosen, 2004; Jerosch-Herold, 2005; Campbell et al., 2013).

It has been assumed that two points are distinguishable from one only when the two points are sufficiently separated to evoke spatially distinct foci of neural activity (Mountcastle and Bard, 1968; Vallbo and Johansson, 1978). Therefore, in the “textbook view” of the 2PD task, two points that fall closely together, for instance within a single afferent receptive field, will evoke only one locus of neural activity and consequently will be misperceived as a single point (Brodal, 2010; Purves et al., 2012). Accordingly, the threshold separation at which neurologically healthy individuals can correctly identify two points has been assumed by many to reflect the size and spacing of cutaneous receptive fields, particularly the innervation density of slowly adapting type-I (SA-1) afferents, the tactile axons that convey fine spatial information (Johnson, 2001).

Nevertheless, the 2PD task has faced serious criticism, because the literature relating 2PD threshold to innervation density is contradictory. As expected of a valid test of spatial acuity, 2PD is indeed reportedly worse on skin sites where SA-1 afferents are more sparsely distributed; for instance, the 2PD threshold is much larger on the forearm than on the fingertips (Weinstein, 1968). Paradoxically, however, healthy participants could perform a two-interval forced-choice (2IFC) 2PD task at approximately 80% accuracy on the fingertip, even when the two-point stimulus was delivered at zero separation (Johnson and Phillips, 1981). This apparently extraordinary spatial resolution is difficult to reconcile with the approximately 1.2 mm center-to-center spacing between fingertip SA-1 receptive fields (Johansson and Vallbo, 1979, 1980; Olausson et al., 2000). For this reason, and because of the large unexplained variation in 2PD thresholds across subjects and studies, investigators have questioned the validity of 2PD as a measure of spatial acuity (Johnson and Phillips, 1981; Johnson et al., 1994; Stevens and Patterson, 1995; Craig and Johnson, 2000; Lundborg and Rosen, 2004).

One plausible explanation for a measured 2PD threshold that falls well under the receptor spacing is that participants are able to exploit a non-spatial cue to perform the 2PD task (Craig and Johnson, 2000). Indeed, two closely spaced points elicit fewer action potentials in the underlying SA-1 afferents than does a single point of equal indentation (Vega-Bermudez and Johnson, 1999). For this reason, perhaps the brain need not discern the spatial profile of the neural activity evoked by a stimulus, but rather only the overall response magnitude (e.g., total number of action potentials in the afferent population), in order to reliably perform the task. If this were the case, participants would be able to infer whether a stimulus consisted of two closely spaced points or one without actually perceiving two distinct points pressing against the skin. As a consequence, the 2PD task would be prone to yield spuriously good spatial acuity measurements, and some sensory deficits would go undetected, underestimated, or inaccurately quantified by 2PD testing, as reported (Van Boven and Johnson, 1994; van Nes et al., 2008).

As others have noted, the continuing popularity of 2PD testing owes largely to the absence of an equally convenient but rigorous alternative task (Lundborg and Rosen, 2004). Here, we investigated one such alternative task, two-point orientation discrimination (2POD), in which the participant must discriminate the orientation (horizontal vs. vertical) of two points of contact. Because the participant is stimulated always with two points, we hypothesized that neural magnitude cues would be absent from this task. The 2POD task would therefore force the participant to rely entirely on the perceived spatial profile of the evoked neural activity, providing a pure measure of spatial acuity. To test our hypothesis, we measured the performance of the same participants on two-interval forced-choice versions of both tasks, on four body sites: fingertip, finger base, palm, and forearm.

## Materials and Methods

### Participants

Twenty-four neurologically healthy participants (18–26 years old, median age 21 years, 14 men, 22 right-handed) were recruited from the McMaster University community. Participants were screened by survey to ensure they did not have conditions that could adversely affect their tactile sensitivity (e.g., diabetes, carpal tunnel syndrome, calluses, or injuries on tested skin areas) or perceptual processing (dyslexia, attention deficit disorder, learning disability, central nervous system disorders) (Grant et al., 1999). Signed, informed consent was obtained from each participant. The McMaster University Research Ethics Board approved all procedures.

### Sensory testing

**Figure 1:**
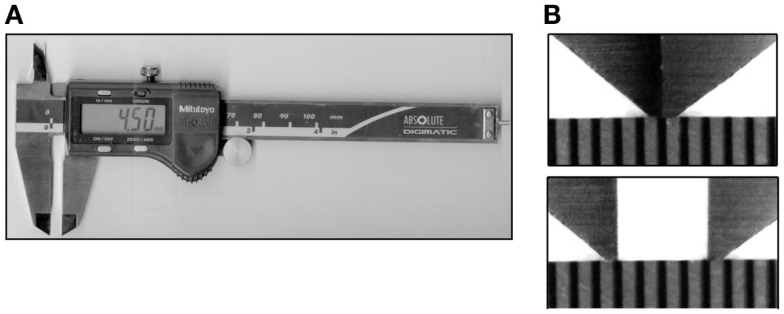
**Calipers**. **(A)** The calipers used in the study, opened to a tip separation of 4.5 mm. **(B)** Magnified images of the caliper tips above a scale with marks at 0.5 mm intervals. Top: closed caliper tips (0 mm separation); bottom: tips opened to 2.0 mm separation.

The participant’s right hand and forearm rested comfortably on a towel spread over a desktop, with the palm facing upwards. A partially open box with a cutout for the arm obscured the participant’s view while leaving the arm visible and accessible to the experimenter. The tactile stimuli were the tip(s) of an Absolute Digimatic calipers (Mitutoyo Corp.) (Figure 1A). The width of each tip was approximately 0.25 mm and the thickness approximately 0.5 mm; thus, when fully closed, the caliper tips formed a 0.5 mm by 0.5 mm square contact surface on the skin (Figure 1B). The experimenter lightly pressed the caliper against the skin, ensuring visually that the skin did not indent so much as to contact the edge of the caliper jaw; estimated skin indentation was ≤2 mm. The participants did not report any discomfort with the application of stimuli. We purposefully used hand-held calipers, rather than automated equipment, in order to reproduce the manual application typically used in clinics.

**Figure 2:**
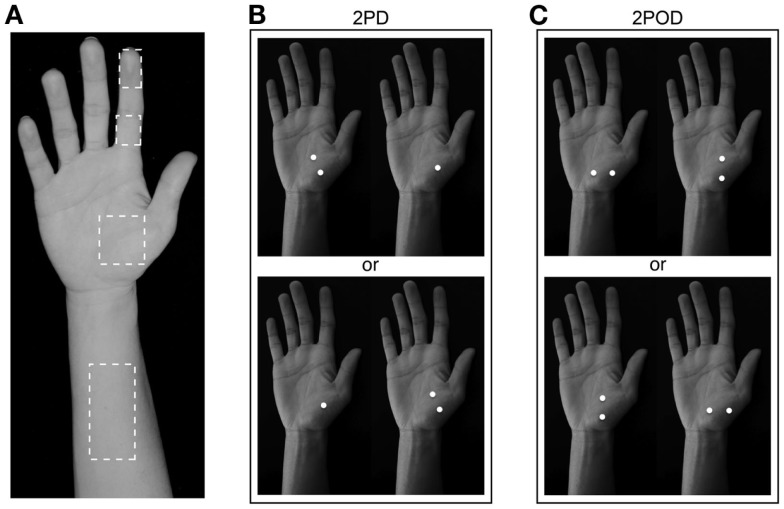
**Two-interval forced-choice perceptual tasks**. **(A)** The four test locations are indicated (dashed outlines): forearm, palm, finger base, and fingertip. **(B)** 2PD task (shown on palm as an example). Participants reported whether the two-point stimulus preceded (upper) or followed (lower) the one-point stimulus. **(C)** 2POD task (shown on palm as an example). Participants reported whether the horizontally oriented two-point stimulus preceded (upper) or followed (lower) the vertically oriented two-point stimulus.

We tested the participants on four skin sites on the right hand and arm: index fingertip pad, index finger base pad, palm (thenar eminence), and volar surface of the forearm (Figure 2A). Each participant was tested with both the 2IFC 2PD task (Figure 2B) and the 2IFC 2POD task (Figure 2C) on every skin site (two tasks × four skin sites = 8 testing blocks of 50-trials each, for a total of 400 trials per participant). One of the 24 possible skin-site orders (four-factorial) was randomly assigned to each of the participants. Following the assigned order, the participant was tested sequentially on the four skin sites, first with one task (testing blocks 1–4), then again in the same order with the other task (testing blocks 5–8). Twelve of the participants were tested first with the 2PD task, and the other 12 first with the 2POD task.

In the 2PD task (Figure 2B), on each trial we indented the calipers approximately 2 mm into the skin surface, once with just one tip (the one-point stimulus) and once with both tips (the two-point stimulus), in randomized order. The two-point stimulus was oriented diagonally (i.e., at ±45-degrees relative to the long axis of the arm, with equal probability). Participants indicated whether they perceived the two-point stimulus before or after the one-point stimulus.

In the 2POD task (Figure 2C), on each trial we indented the calipers approximately 2 mm into the skin surface, once with two points oriented parallel (vertical) and once with two points oriented perpendicular (horizontal) to the long axis of the arm, in randomized order. Participants indicated whether they perceived the horizontally oriented points before or after the vertically oriented points.

In all tests, participants registered their responses by pressing one of two buttons on a wireless remote (Kensington, model 33374) held with the left hand. Feedback was not provided. During all tests, pink noise was played over computer speakers (Noise X 1.1 for MacIntosh, Blackhole Media Co.) to mask any potential auditory cues associated with the adjustment of the calipers.

### Adaptive psychophysical procedure and Bayesian parameter estimation

**Figure 3:**
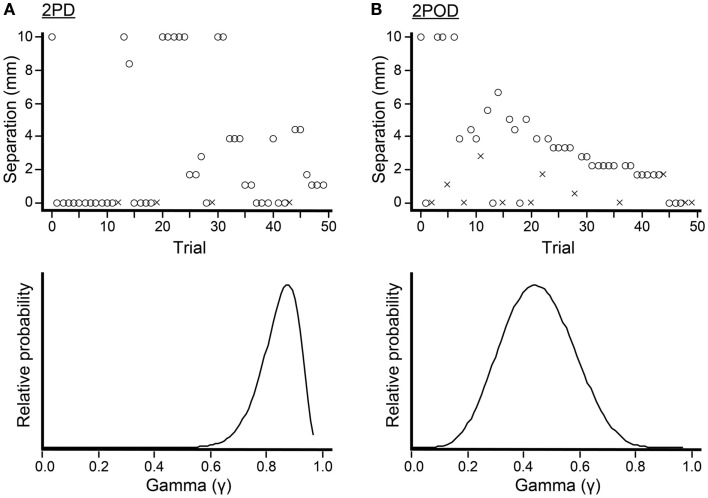
**Bayesian adaptive testing procedure**. Plots illustrate the trial-by-trial performance (upper) and gamma parameter posterior density (lower) for experiments done on the finger base of one participant. **(A)** 2PD task. **(B)** 2POD task. Crosses represent incorrect responses; circles, correct responses. Note that, at zero mm tip separation, this participant answered correctly on 21/25 = 84% of 2PD trials, compared to 6/13 = 46% of 2POD trials. The Bayesian adaptive procedure does not follow a preset stimulus sequence or simple staircase algorithm, but rather selects the separation on each trial that is expected to provide the most information regarding the shape of the participant’s psychometric function.

To conduct the 2PD and 2POD tasks, we used a Bayesian adaptive algorithm, modified from Kontsevich and Tyler (1999), which we programed in LabVIEW 9 (National Instruments) for Macintosh. The algorithm efficiently estimated a participant’s psychometric function (proportion of correct responses at each tip separation, *x*) by choosing on each trial the two-point separation that was predicted to yield the most information in light of the participant’s previous responses (expected entropy minimization). A computer monitor (out of the participant’s view) displayed that tip separation to the investigator, who adjusted the calipers to select the instructed tip separation with a precision of 0.1 mm (Figure 3). For fingertip, finger base, and palm testing, the computer algorithm chose from among 19 tip separations, equally spaced between 0 and 10 mm (i.e., 0, 0.6, 1.1, 1.7, … 10.0 mm). For forearm testing, the algorithm chose from among 19 tip separations, equally spaced between 0 and 45 mm (i.e., 0, 2.5, 5.0, 7.5, … 45.0 mm).

Our algorithm considered a set of 500,000 possible psychometric functions for the participant’s performance on a given testing block, parameterized as Weibull functions (Klein, 2001; Wichmann and Hill, 2001):

$$\Psi_{\text{a,b,}\gamma }\left(x\right)=\gamma +\left(1-\gamma -\delta \right)\left(1-2^{-{\left(\text{x/a}\right)}^{\text{b}}}\right)$$

Each psychometric function was characterized by four parameters: γ, the proportion correct at zero tip separation; *a*, the tip separation at which the proportion correct was midway between that at zero tip separation and that at infinite separation; *b*, the function slope; and δ, the lapse rate. The set of possible psychometric functions consisted of all possible combinations of γ (100 equally spaced values, ranging from 0.01 to 0.99), *a* (100 equally spaced values, ranging from 0.01 to 60 mm), and *b* (50 equally spaced values, ranging from 0.1 to 10); the lapse rate, δ, was set to 0.02. We applied a uniform prior probability distribution over psychometric functions, *P*(Ψ_a,b,γ_) = 1/500,000.

From the participant’s set of correct and incorrect responses, {*r*_i_}, on the 50 trials within a testing block, the algorithm calculated the posterior probability of each psychometric function, *P*(Ψ_a,b,γ_ | {*r*_i_}), as well as marginal posterior densities and maximum *a posteriori* estimates (modes) for each of the three free parameters: γ, *a*, and *b*. To obtain finer resolution in this offline analysis, we used 100 values for each parameter, with the following ranges: *a* (0.01–10 mm for fingertip and finger base; 0.01–50 mm for palm and forearm), *b* (1–10), γ (0.01–0.97). We took the mode of each parameter’s marginal posterior density as the best-estimate for the parameter’s value.

Additionally, we calculated the probability of the participant’s data given random guessing on every trial, divided by the probability of the data given a psychometric function. We obtained the latter probability by integrating over the space of all psychometric functions, weighting the probability of the data given each function by the prior probability of that function. Thus, the formula for this ratio was:

$$BF=\frac{{\left(0.5\right)}^{50}}{\underset{a,b,\gamma }{∭}P\left(\{r_{i}\}|\Psi_{a,b,\gamma }\right)P\left(\Psi_{a,b,\gamma }\right)d_{a}d_{b}d_{\gamma }}$$

This ratio, a Bayes’ Factor (BF) for guessing, reaches 1 only if a participant’s responses are as likely to occur from pure guessing as from a psychometric function. Thus, if a participant’s BF (rounded to the nearest integer) was ≥1 on any testing block, we eliminated all of the participant’s data from subsequent analyses. This procedure ensured that our analyses considered data only from participants who were consistently concentrating during the sensory testing. Out of our original pool of 24 participants, 5 were eliminated on this basis.

To obtain a best-estimate of a participant’s probability of correct responding as a function of tip separation, *p*_c_(*x*), we integrated over the psychometric function posterior distribution the proportion correct predicted by each function:

$$p_{\text{c}}(x)=\underset{\text{a},\text{b},\gamma }{∭}\Psi_{\text{a},\text{b},\gamma }(x)P\left(\Psi_{\text{a},\text{b},\gamma }|\{r_{\text{i}}\}\right)d_{\text{a}}d_{\text{b}}d_{\gamma }$$

To obtain the mean performance across participants on each body site, we averaged *p*_c_(*x*) across participants.

We determined for each testing block the tip separation (*x*_95%_) at which the participant responded correctly with 95% probability. The probability of a particular *x*_95%_ value was calculated by summing the posterior probabilities of all psychometric functions that crossed 95% within ±0.05 mm of that value. Repeating this procedure for all possible *x*_95%_ values, we obtained a probability distribution over *x*_95%_, the mode of which we report as our best-estimate of the participant’s 95%-correct threshold.

### Data analysis

Analyses of variance (ANOVA), *t*-tests, chi-square tests, and correlations were performed with SPSS v20 (IBM Corp.) for MacIntosh, using an alpha-level of 0.05. We report two-tailed *p*-values. The ANOVA model was full-factorial type III sum-of-squares.

## Results

### 2POD but not 2PD approached 50%-correct at zero tip separation

**Figure 4:**
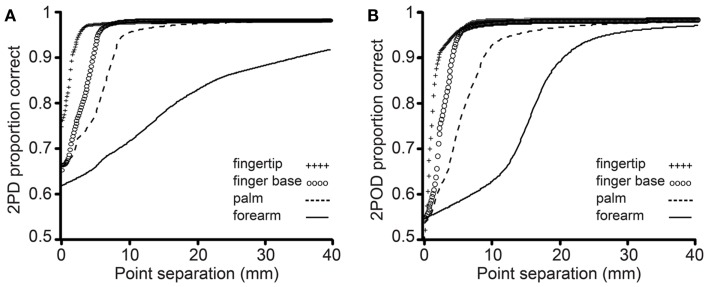
**Mean performance by task and body site**. Proportion correct versus caliper tip separation on **(A)** 2PD and **(B)** 2POD. Data points are means across all participants. For illustration purposes, all curves have been extended to 40 mm on the *x*-axis.

The mean performance for each task at each body site is shown in Figure 4. In accord with our prediction, the psychometric functions for the two tasks clearly differed in their percent-correct performance at zero tip separation, with performance being close to chance (50%-correct) for the 2POD but not the 2PD task.

**Figure 5:**
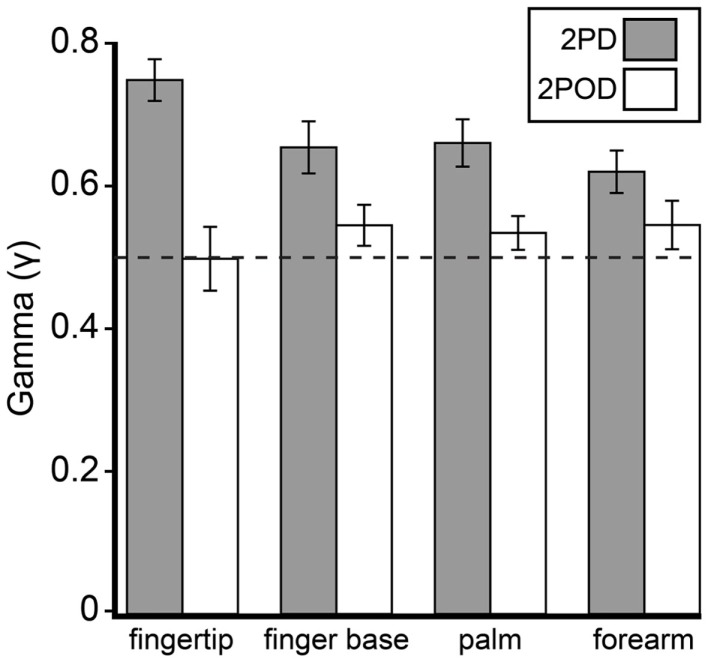
**Mean γ-parameters by task and body site**. Gray bars, mean 2PD γ; white bars, mean 2POD γ. Error bars: ±1 SE. Dotted line: γ = 0.5.

This observation was confirmed by an analysis of the psychometric function gamma parameter (performance at zero tip separation) (Figure 5). A two-way (task × body site) repeated-measures ANOVA, with γ as the dependent variable, revealed a highly significant effect of task (*F* = 26.35, *p* < 0.001) with no significant effect of body site (*F* = 0.60, *p* = 0.618) or task-by-body site interaction (*F* = 2.52, *p* = 0.068). Four *post hoc* one-sample t-tests with Bonferroni correction revealed that the mean 2PD γ value was significantly above 0.5 on all body sites (all corrected *p*-values < 0.005). In stark contrast, the mean 2POD γ value did not differ significantly from 0.5 on any body site (all corrected *p*-values > 0.5). In contrast to the gamma parameter, the *a* and *b*-parameters did not differ significantly between tasks (separate two-way repeated-measures ANOVAs, *p* = 0.063 and 0.561 for main effects of task on *a* and *b*-parameters, respectively).

### 2POD but not 2PD consistently yielded a 75%-correct threshold

Having found that performance at zero tip separation differed dramatically between the tasks, we next turned our attention to the participants’ performance at non-zero tip separations. The 75%-correct threshold is a commonly reported psychophysical performance measure; for the experiments reported here, this threshold would be the tip separation at which a participant’s psychometric function crossed the 0.75 mark. We were unable to compare the two tasks on this basis, however, because the 2PD task often failed to produce a measurable 75%-correct threshold.

Remarkably, the gamma parameter values characterizing participant performance on the 2PD task tended to be so large that only 5 of 19 participants had a measurable 75%-correct 2PD threshold (i.e., gamma ≤75%) on all skin sites. In contrast, 15 of 19 participants had measureable 75%-correct 2POD thresholds at all skin sites. Indeed, of the 76 2PD testing blocks (19 participants × 4 skin sites), only 53 resulted in measurable 75%-correct thresholds. In contrast, 72 of the 76 2POD testing blocks resulted in measurable 75%-correct thresholds. These differences between tasks were highly significant (participant count comparison: chi-square = 10.56, *p* = 0.001; total testing block count comparison: chi-square = 16.26, *p* < 0.001). Thus, the 2PD task, unlike the 2POD task, often failed to yield a conventional threshold measure.

### 2POD and 2PD had similar 95%-correct thresholds that correlated with receptor spacing

**Figure 6:**
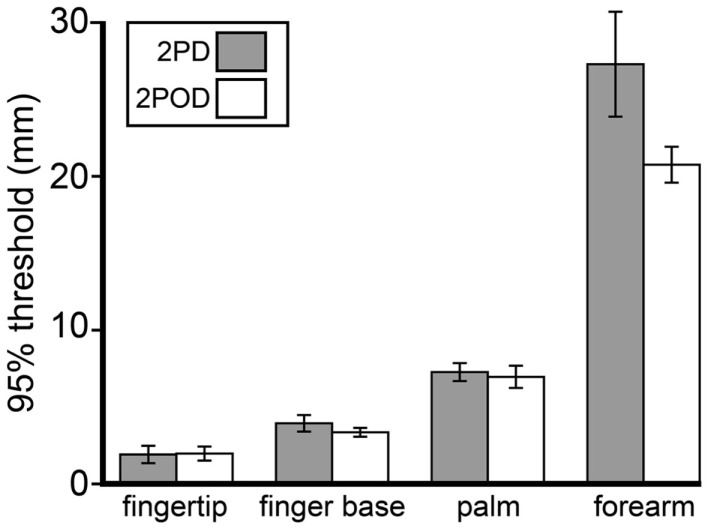
**Mean 95%-correct thresholds by task and body site**. Gray bars, mean 2PD 95%-correct thresholds; white bars, mean 2POD 95%-correct thresholds. Error bars: ±1 SE.

Because we were unable to obtain a consistent 2PD 75%-correct threshold, we chose instead to compare 95%-correct thresholds, which were measurable on all testing blocks. Interestingly, although performance at small tip separations differed significantly between tasks, performance on the tasks converged as tip separation increased. In particular, the 95%-correct threshold did not differ significantly between tasks (Figure 6). A two-way (task × body site) repeated-measures ANOVA, with 95%-correct threshold as the dependent variable, showed a highly significant effect of body site (*F* = 106.50, *p* < 0.001) but no significant effect of task (*F* = 3.86, *p* = 0.065).

**Figure 7:**
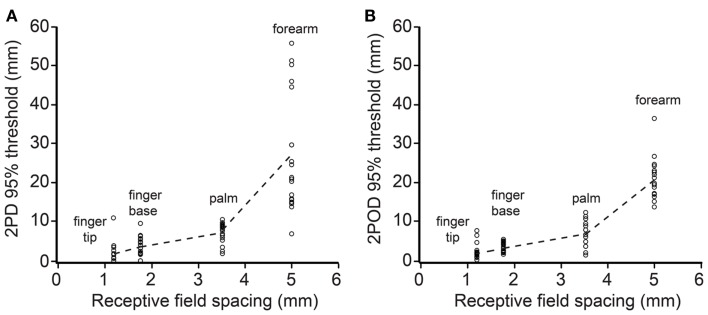
**95%-correct thresholds versus receptor spacing**. Participants’ 95%-correct thresholds for 2PD **(A)** and 2POD **(B)** plotted against estimated SA-1 receptive field spacing (Johansson and Vallbo, 1979; Olausson et al., 2000): fingertip (1.20 mm), finger base (1.77 mm), palm (3.53 mm), forearm (5.00 mm). Data points show individual participant performance; dashed lines connect group means.

We next investigated how 2PD and 2POD 95%-correct thresholds related to the distribution of tactile receptors. For each participant, we correlated the 95%-correct thresholds with the estimated receptive field spacing of human SA-1 afferents (Johansson and Vallbo, 1979; Olausson et al., 2000). The 95%-correct performance on both tasks correlated significantly with estimated receptive field spacing (mean Pearson’s *r* correlation coefficients: 2PD: *r* = 0.906, *p* < 0.001; 2POD: *r* = 0.915, *p* < 0.001) (Figure 7).

## Discussion

The 2PD task is widely used clinically (Dellon, 1981; American Society for Surgery of the Hand, 1983; Van Boven and Johnson, 1994; Lundborg and Rosen, 2004; Jerosch-Herold, 2005; Campbell et al., 2013) and has been used also in several research laboratories to characterize tactile spatial acuity in healthy populations (Godde et al., 2000; Kennett et al., 2001; Dinse et al., 2006; Boles and Givens, 2011). Nevertheless, our results confirm that the 2PD threshold is not a pure measure of spatial acuity. The data support the use of an equally convenient alternative task – 2POD. Unlike 2PD, 2POD performance approaches chance levels as tip separation approaches zero, as expected of a rigorous measure of spatial acuity.

### 2PD performance benefits from a non-spatial cue

Our findings support and extend upon a previous literature revealing that the 2PD task presents a non-spatial cue. Like Johnson and Phillips (1981), who conducted 2PD testing on the fingertip, we found that participants could reliably discriminate between a single point and two points at zero separation. On the fingertip, finger base, palm, and forearm, the mean 2PD γ value was significantly above 0.5, indicating that participants were able to perform correctly even at zero tip separation. Thus, 2PD performance is starkly inconsistent with the known spatial distribution of SA-1 mechanoreceptive afferents (Johansson and Vallbo, 1979, 1980; Olausson et al., 2000). We conclude that the 2PD task presents a non-spatial cue, allowing participants to infer the presence of two points without distinctly perceiving them.

**Figure 8:**
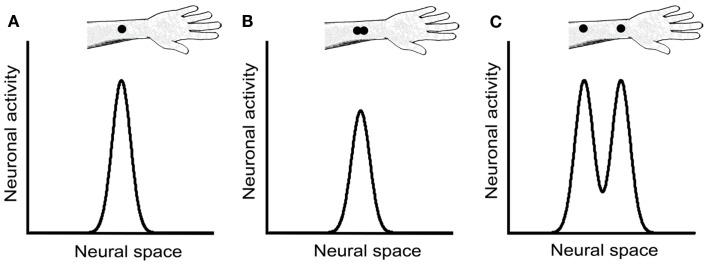
**Neural response magnitude cues in the 2PD task**. The three panels show hypothetical activity profiles of a population of central somatosensory neurons in response to three stimulus configurations: **(A)** a single point, **(B)** two closely spaced points, and **(C)** two points separated by a greater distance. We assume that the activity of central neurons reflects approximately that of the SA-1 afferents, described in Vega-Bermudez and Johnson (1999). In the textbook view of the 2PD task, the stimulus configurations illustrated in **(A)** and **(B)** would be indistinguishable from one another, because both configurations result in a single peak of neural activity. However, the neurophysiological data (Vega-Bermudez and Johnson, 1999) suggest that the population response in **(B)** is of lower magnitude than in **(A)**, a cue that allows the participant to distinguish **(A)** from **(B)** by non-spatial means. In **(C)**, the two activity peaks are indeed distinguishable spatially; in addition, because each activity peak in **(C)** has equal height to the single peak in **(A)**, the total population response in **(C)** is greater that in **(A)**, giving rise to another magnitude cue.

We concur with Craig and Johnson (2000) that a likely non-spatial cue in the 2PD task is a response magnitude cue: due either to skin mechanics or to neural interactions among branches of individual afferent fibers, two closely spaced stimulus points elicit fewer action potentials in the underlying afferents than does a single-point of equal indentation (Vega-Bermudez and Johnson, 1999). For instance, when a one-point stimulus over an SA-1 receptive field center is compared to a two-point stimulus consisting of that same point plus another at 1 mm distance, the two-point stimulus elicits on average about 30% fewer action potentials. A similar effect, though weaker in magnitude, is observed when neither point overlies the center of the receptive field (Vega-Bermudez and Johnson, 1999). Thus, by merely detecting the total number of action potentials elicited in the afferent population rather than the spatial profile of neural activity, a participant could infer whether the stimulus contained one point or two (Figures 8A,B).

We note that a magnitude cue will also exist, in the opposite direction, at somewhat larger tip separations, where interactions between stimuli are not expected at the single-neuron level. For instance, a two-point stimulus at 1 cm separation should elicit about twice the number of action potentials in the afferent population as would a one-point stimulus of equal indentation, because the two-point stimulus will activate about twice as many neurons (Figures 8A,C). Therefore, the 2PD task is apparently beset with magnitude cues at all tip separations.

An additional non-spatial cue that might sometimes accompany the 2PD task is a temporal cue: if the investigator fails to apply the two points simultaneously, the participant may perceive two contacts that are distinct in time. In this case, the participant could infer that two points touched the skin, even when unable to distinguish the points spatially. A limitation of any manual stimulus application method is that exact simultaneity is not achievable. Because humans are able to distinguish temporal delays between tactile stimuli of approximately 10 ms (Gescheider, 1967; Gescheider et al., 2003), any delay of this duration or longer between the two points of contact could produce a perceptible temporal cue. We note, however, that even when the 2PD task was conducted with an automated apparatus that touched the two tips against the skin with less than 2 ms delay, performance was approximately 80%-correct at zero tip separation (Johnson and Phillips, 1981). Thus, a temporal cue, while plausibly facilitating 2PD task performance under manual stimulus delivery, is unlikely to account for the extraordinary performance of participants at zero tip separation.

An alternate explanation for above-chance 2PD performance at zero tip separation, put forth by Stevens and Patterson (1995), is that participants make use of a length cue: two apposed points might feel longer than a single point. However, we believe it unlikely that our participants could detect the 0.25 mm difference in length between our single-point stimulus and the two apposed points. In a length discrimination experiment using raised edges of either 0.5 or 5 mm baseline length, Stevens and Patterson (1995) reported that on the fingertip the average adult participant could distinguish with 71% accuracy edges that differed by 0.8–0.9 mm in length. This length discrimination threshold is consistent with the estimated SA-1 receptive field spacing on the fingertip of approximately 1 mm (Johansson and Vallbo, 1979, 1980; Olausson et al., 2000). The implication of this finding is that the 2PD task would present a perceptual length cue at zero tip separation on the fingertip whenever the individual points have a size of approximately 0.8 mm or more. This would seem to rule out a length cue in the present study, as our point stimulus had a width of approximately 0.25 mm. Furthermore, to be detectable on the finger base, palm, and forearm, which have lower receptor densities than the fingertip, the length difference would presumably need to be much larger than 0.8 mm. Nevertheless, our participants performed significantly above chance at zero tip separation on those body sites as well.

### 2PD performance reflects both spatial and non-spatial information

Because it is contaminated by one or more non-spatial cues, the 2PD task is prone to yield spuriously good performance. Consequently, tactile spatial deficits – particular if not severe – may be undetected or underestimated by 2PD testing. For instance, van Nes et al. (2008) reported that 2PD testing detected mild polyneuropathy caused by diabetes mellitus, chronic inflammatory demyelinating polyneuropathy, Guillain-Barré syndrome, uremia, and other causes, with a sensitivity of only 28%. Similarly, Van Boven and Johnson (1994) found that following elective mandibular surgery that injured but did not transect the inferior alveolar nerve, 2PD on the lip returned to normal levels much earlier in the course of recovery than did grating orientation performance, a rigorous measure of spatial acuity (see below). The authors argue that, owing to the presence of non-spatial cues, 2PD grossly overestimated the initial recovery of tactile spatial function.

Despite the presence of non-spatial cues, it would be an overly critical indictment to conclude that 2PD conveys no information regarding a patient’s spatial acuity. It seems clear that spatial as well as non-spatial cues influence 2PD task performance, particularly at larger tip separations. Presumably for this reason, more severe injuries, such as nerve transections, do result in lasting elevation of 2PD thresholds despite the return of tactile sensitivity as measured by monofilament testing (Rosen et al., 2000; Jerosch-Herold, 2003). Nerve transection, unlike nerve crush, is thought to result in the misdirection of sensory axons during re-innervation; the shuffling of these axons causes severe deficits in spatial acuity (Van Boven and Johnson, 1994; Rosen et al., 2000), thereby elevating the 2PD threshold.

Among the neurologically healthy participants tested here, fewer than half had measurable 75%-correct 2PD thresholds on the four skin sites; due presumably to non-spatial cues, performance did not consistently drop below 75%-correct even at zero tip separation. Nevertheless, the 2PD performance of all participants did fall below 95%-correct at small tip separations. Analyzing participants’ 95%-correct thresholds on the four body sites, we found that they correlated with mean receptive field spacing. This result is in keeping with previous reports that 2PD performance worsens on skin areas with sparser receptor distribution (Weinstein, 1968). Furthermore, the 95%-correct thresholds on the 2PD task did not differ significantly from those on the 2POD task. Presumably, at larger tip separations when distinct points are more reliably perceptible, participants do make use of the spatial pattern of the afferent population discharge.

For researchers who wish to use the 2IFC 2PD task, these results might suggest the adoption of the 95%-correct threshold as a valid performance measure. Nevertheless, we caution that the accurate estimation of a 95%-correct threshold is difficult. Conducting computer simulations of sensory tests using the method of limits, for instance, we found that the test-retest variance of the 95%-correct threshold estimate was consistently – and often considerably – greater than that of the 75%-correct threshold estimate. This difference owes to the shallower slopes of the psychometric functions (Figure 4) as they near the upper asymptote, which translates into a greater uncertainty in the *x*-axis value of the estimate, caused by any uncertainty in the %-correct measurement (Zuberbühler, 2002). Rather than attempting to estimate a 95%-correct threshold, we suggest that clinicians and researchers simply set aside the 2PD task and replace it with one that ensures a more purely spatial measure of acuity.

In this study, we conducted a 2IFC version of the 2PD task in order to most accurately assess the presence of non-spatial cues. In the 2IFC version, because a single-point and a two-point stimulus are presented on each trial, the participants are able to directly compare the neural responses that occur in the two configurations. Participants may therefore rather quickly become aware of non-spatial cues in this version of the task. A commonly used alternative version of the task employs single-interval trials. In each trial, the participant is stimulated just once, with either one or two-points, and asked to identify the configuration. This single-interval version of the task, though subject to the effects of response criteria (Gescheider, 1997; MacMillan and Creelman, 2005), may in fact be preferable to the 2IFC version, because with appropriate instruction the participant can be encouraged to respond “two” only when two distinct points are clearly perceived (Kalisch et al., 2007). The single-interval task may therefore mitigate the effect of neural magnitude cues on performance, thereby yielding a more purely spatial measure of acuity. In this regard, we note that the average single-interval 2PD 50% correct threshold obtained by Kalisch et al. (2007) from the right index fingers of untrained participants was approximately 1.6 mm, a tip separation that presented in our 2IFC 2PD task would yield on average 85% correct performance (see Figure 4A). Based on our finding that the 2PD and 2POD tasks yield similar performance at large tip separations, we suspect that the thresholds measured by Kalisch et al. (2007) indeed reflect primarily the participant’s spatial acuity. In general, the single-interval 2PD task, combined with instructions to participants to adopt an appropriately conservative response criterion, may produce the most reliable spatial acuity data achievable with the 2PD task.

### 2POD is a rigorous and convenient measure of tactile spatial acuity

Unlike the 2PD task, the 2POD task involves the spatial discrimination of orientation, with two points always presented. Thus, we reasoned that the 2POD task would avoid the non-spatial cues that plague the 2PD task: the neural population response magnitude should be the same, on average, for the two orientations, and a temporal delay between the two points of contact, if present, would not compromise the task; to perform successfully, the participant would still need to discern the orientation of the points. Therefore, we predicted that 2POD performance would approach chance as the tip separation approached zero. Our results confirmed this prediction.

To our knowledge, we are the first to propose the exact version of the 2POD task described here, though Stevens and colleagues used similar tasks (Stevens and Patterson, 1995; Stevens et al., 1996) and Weber himself explored two-point perception in the horizontal compared to the vertical orientation (Weber, 1996). In Stevens and Patterson (1995), a pair of longitudinal two-point stimuli and a pair of two-point stimuli of non-matching orientations (longitudinal and transverse) were presented on every trial; the participant was asked to identify which interval had the non-matching pairs. In Stevens et al. (1996), a single two-point stimulus was given in either longitudinal or transverse orientation, and the participant was asked to identify the orientation. Some participants in Stevens and Patterson (1995) performed correctly at zero tip separation, perhaps because relatively large caliper tips (0.44 mm each) permitted the perception of orientation even when fully closed. To prevent this, we recommend that the 2POD task be performed with caliper tips of approximately 0.25 mm diameter.

The 2POD task that we have used combines the rigor of a gold standard in tactile spatial acuity testing, the grating orientation task, with the convenience of the 2PD task. In the grating orientation task, participants attempt to discern the orientation (typically, horizontal or vertical) of square-wave gratings with equal ridge and groove width. Groove width is reduced to make the task more difficult, or increased to make it easier. Acuity is measured as the groove width whose orientation the participant can discern with a particular probability (e.g., 75%-correct). Whether a grating is applied horizontally or vertically, it is expected to elicit on average the same afferent population discharge magnitude; only the spatial structure of the population discharge varies. Therefore, to perform the task correctly the participant must discern the spatial pattern of afferent activity, rendering this a rigorous test of tactile spatial acuity (Johnson and Phillips, 1981; Gibson and Craig, 2002, 2006). The similarity to the 2POD task is clear.

While tactile research laboratories such as ours make extensive use of the grating orientation task (Goldreich and Kanics, 2003; Goldreich et al., 2009; Peters et al., 2009; Wong et al., 2011a,b, 2013), we recognize that the task has certain practical disadvantages, particularly as concerns the clinical setting. Among these is that each grating must be prefabricated; consequently, the variable of interest, groove width, cannot be adjusted outside a pre-determined range. This is particularly problematic if one wishes to test patients who may have atypical spatial acuity due to neurological damage. The 2POD task does not suffer from this practical inconvenience. Rather, like the 2PD task, the 2POD task is remarkably flexible in requiring only a single tool (calipers) that is easily adjustable during testing.

### Recommendations for future studies and for clinical practice

**Figure 9:**
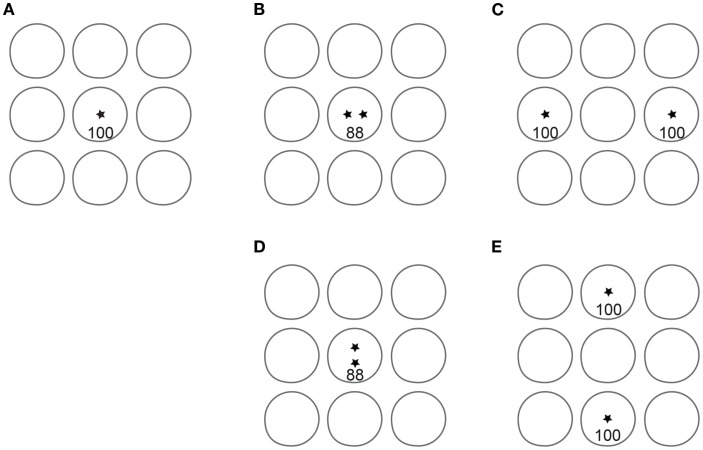
**Advantage of 2POD over 2PD for tactile spatial acuity assessment**. Each panel depicts idealized circular receptive fields of nine SA-1 afferents; for clarity, only non-overlapping fields are shown. Asterisks represent point stimuli. In 2PD, the participant attempts to distinguish between a single point **(A)** and two points separated by some distance, e.g., **(B)** or **(C)**. For illustration, we assume that a single point evokes 100 action potentials per second in the central SA-1. When two points fall within the same receptive field **(B)**, they evoke fewer action potentials than the single point. For instance, two points at 1 mm separation evoke on average 88% the firing rate of a single point (Vega-Bermudez and Johnson, 1999). Thus, the participant can distinguish one from two points based on the number of action potentials (magnitude cue), even when the two points cannot be individually perceived. When separated by a greater distance **(C)**, the two points can be perceived, because they fall within separate receptive fields (spatial cue). In addition, the magnitude cue has reversed direction, as the total number of action potentials in the two-point condition (200) is twice that in the one-point condition. Thus, the two-point task conveys spatial information at larger separations but is contaminated by a magnitude cue at all separations. In 2POD, the participant attempts to distinguish between two points separated horizontally and two points separated vertically by the same distance: **(B)** vs. **(D)**, or **(C)** vs. **(E)**. These stimuli evoke an equal number of action potentials, eliminating the magnitude cue and forcing the participant to rely on purely spatial information. When the points fall within a single receptive field, as in **(B)** and **(D)**, their orientation is indistinguishable. When the points fall within distinct receptive fields, as in **(C)** and **(E)**, their orientation is distinguishable.

In conclusion, our data confirm that the 2IFC 2PD task is contaminated by one or more unintended non-spatial cues that result in inflated spatial acuity reports. An alternative task, 2POD, provides a rigorous measure of spatial acuity. The advantage of 2POD over 2PD as a measure of spatial acuity is summarized in Figure 9.

We have performed the 2POD task using vertically and horizontally (i.e., longitudinally and transversely) oriented stimuli. One recommendation for future studies and for clinical practice would be to use oblique (e.g., ±45-degree) orientations. The use of oblique stimuli would offer two practical advantages. First, it would permit greater tip separations. On the digits and limbs, the maximum tip separation in the vertical-horizontal 2POD task is limited to the width of the body part, a constraint that is overcome by the use of oblique stimuli. Second, the use of oblique stimuli would prevent magnitude cues that might arise from receptive field anisotropy. A majority of receptive fields on the fingers and palm reportedly are elongated rather than circular; furthermore, roughly two-thirds of the elongated fields are oriented longitudinally with respect to the arm (Johansson and Vallbo, 1980). Perhaps for this reason, performance anisotropy has been reported on several body areas, in a variety of tactile acuity tests (Essock et al., 1992; Stevens and Patterson, 1995; Gibson and Craig, 2005), beginning with the report by Weber himself that 2PD acuity was better when the tips were aligned transversely (Weber, 1996). The use of oblique stimuli should prevent performance anisotropy caused by alignment of the two-point configuration in parallel or orthogonal to the average receptive field orientation.

Given its evident advantages, we recommend that 2POD replace 2PD testing in the clinic and in research settings. Additional studies should be carried out to further validate the 2POD task by measuring inter-rater and test-retest reliability and by comparing 2POD with grating orientation thresholds in neurologically healthy participants and in patients. Our laboratory has previously shown that grating orientation thresholds correlate with fingertip surface area (Peters et al., 2009), suggesting that receptive fields are more widely spaced in larger fingers. As an exploratory analysis, we checked for this effect in the current 2PD and 2POD data, but not surprisingly, we observed no significant correlations between finger size and performance on either task in our relatively small participant sample. In analogy with previous grating orientation studies, we predict that, with sufficiently large sample sizes (Peters et al., 2009) or with trained participants (Wong et al., 2013), 2POD performance will also be found to correlate with finger size.

Although we have used adaptive psychophysical data collection methods and mathematical analyses in order to evaluate the 2POD and 2PD tasks, we suggest that more practical, less elaborate procedures be used in the clinic. To facilitate the use of the task for clinical purposes, we recommend that the patient be stimulated with 10 or 20 2POD 2IFC trials at each of several tip separations. A plot could then be made of the number of correct responses at each separation. The interpolated tip separation corresponding to 75%-correct could be reported as the patient’s spatial acuity. Alternatively, for greater convenience and to reduce testing time, a single-interval 2POD task could be used, in which the participant is stimulated just once on each trial, and attempts to identify the stimulus orientation; we favor the 2IFC testing protocol, however, to prevent possible criterion effects (Gescheider, 1997). For equipment, we recommend the use of adjustable calipers with pointed tips not exceeding 0.25 mm width and 0.5 mm thickness. One such device is the Absolute Digimatic caliper (Mitutoyo Corp.) used in this study; many similar devices are available from Starrett Co., Digital Measurement Metrology, Inc., and other companies. The cost of these calipers ranges from under $20 to over $100, depending on their material and precision.

## Conflict of Interest Statement

The authors declare that the research was conducted in the absence of any commercial or financial relationships that could be construed as a potential conflict of interest.
